# Peripheral Visual Reaction Time Is Faster in Deaf Adults and British Sign Language Interpreters than in Hearing Adults

**DOI:** 10.3389/fpsyg.2017.00050

**Published:** 2017-02-06

**Authors:** Charlotte J. Codina, Olivier Pascalis, Heidi A. Baseler, Alexandra T. Levine, David Buckley

**Affiliations:** ^1^Academic Unit of Ophthalmology and Orthoptics, School of Medicine and Biomedical Science, The University of SheffieldSheffield, UK; ^2^Laboratoire de Psychologie et NeuroCognition, Université de Grenoble AlpesGrenoble, France; ^3^Centre for Neuroscience, Hull York Medical School, University of YorkYork, UK; ^4^Department of Psychology, University of YorkYork, UK

**Keywords:** deafness, reaction times, accuracy, British Sign Language, visual attention, peripheral vision

## Abstract

Following auditory deprivation, the remaining sense of vision has shown selective enhancement in visual cognition, especially in the area of near peripheral vision. Visual acuity is poor in the far periphery and may be an area where sound confers the greatest advantage in hearing persons. Experience with a visuospatial language such as British Sign Language (BSL) makes additional demands on the visual system. To test the different and separable effects of deafness and use of a visuo-spatial language on far peripheral visual processing, we investigated visual reaction times (RTs) and response accuracy to visual stimuli, between 30° and 85° along the four cardinal and four inter-cardinal meridians. We used three luminances of static, briefly illuminated stimuli in visually normal adults. The cohort tested included profoundly congenitally deaf adults (*N* = 17), hearing fluent BSL users (*N* = 8) and hearing non-signing adults (*N* = 18). All participants were tested using a peripheral forced choice paradigm designed previously to test deaf and hearing children (Codina et al., 2011a). Deaf adults demonstrated significantly faster RTs to all far peripheral stimuli and exceeded the abilities of both signing and non-signing hearing adults. Deaf adults were significantly faster than BSL interpreters, who in turn were significantly faster than hearing non-signing adults. The differences in RT demonstrated between groups were consistent across all visual field meridians and were not localized to any one region of the visual field. There were no differences found between any groups in accuracy of detecting these static stimuli at any retinal location. Early onset auditory deprivation appears to lead to a response time visual advantage in far peripheral responses to briefly presented, static LED stimuli, especially in the right visual field. Fluency in BSL facilitates faster visuo-motor responses in the peripheral visual field, but to a lesser extent than congenital, profound deafness.

## Introduction

Human peripheral visual perception is affected by sensory, developmental, and environmental experience. The visual system has inherent plasticity, peripheral vision in particular showing a high capacity for plasticity and the potential for peripheral visual plasticity has been previously underestimated (Burnat, 2015). Both the peripheral retina and the magnocellular visual pathway have emergent, immature topographies that may facilitate high levels of visual plasticity throughout life. Far peripheral vision plays a crucial role in monitoring the environment, especially in the absence of sound.

Several visual changes have been noted in association with deafness. Bosworth and Dobkins (2002) showed that deaf adults performed significantly better to peripheral, but not central visual stimuli. Proksch and Bavelier (2002) used a visual search paradigm to report that deaf individuals had greater visual attentional resources in the visual periphery, and less in central vision. Neville and Lawson (1987a,b) found enhanced attention to the visual periphery in a motion decision task, coordinated with event-related potential (ERP) responses from the occipital cortex of deaf participants to peripheral stimuli. Bavelier et al. (2001) by means of fMRI, detected greater recruitment of the motion selective area V5/MT for deaf participants when they attended peripherally rather than centrally. Fine et al. (2005) found that fMRI responses to visual stimuli were uniquely represented in the auditory cortex of deaf participants, and this effect was not seen in adult participants who were children of deaf adults (CODAs), signing from birth. Bavelier et al. (2006) summarized that not all aspects of vision are improved in deaf individuals—deaf adults showing slower reactions in central visual cognitive tasks (Proksch and Bavelier, 2002). Bavelier et al. (2006) argue that selective visual changes occur which compensate for those aspects of vision that would normally benefit from the combined auditory and visual inputs. In line with this, Codina et al. (2011b) found altered retinal ganglion cell layer distribution to support peripheral vision, and increased retinal ganglion cell number and structural changes correlated with increased peripheral vision performance. Deafness therefore has a differential influence on both the structure and behavior of the remaining senses such as vision.

It is not only deafness that has been shown to promote a peripheral visual advantage in humans. Habitual playing of computer games has been linked with improved localization of a peripheral target amongst distractors (Green and Bavelier, 2003), and increased visual field (VF) area Buckley et al. (2010). Memmert et al. (2009) found specific visual attention improvements between athletes and non-athletes when the stimuli were most similar to their practiced sport. Muiños and Ballesteros (2013) reported that karate athletes were faster at localizing peripheral visual stimuli than non-athletes. They suggested that the rapidity of response in their athletes may be due to the suddenly appearing, peripherally attended, opponent maneuver. Different patterns of visual skills may result in specifically trained motor responses to peripheral visual stimuli. There is consensus amongst several authors that the visual differences found between athletes and non-athletes are not in the “hardware” of functional visual pathway changes, but in the “software,” using perception and visual-cognitive processes more efficiently, employing skill utilizing strategies made effective with practice (Abernethy et al., 1994; Muiños and Ballesteros, 2013). Schubert et al. (2015) conducted a detailed investigation on the training effects of video gaming on visual attention. They found that video gamers showed increased visual processing speeds in the lower aspects of the VF. The video gamers did not change their attention allocation strategy, with high speed processing demonstrated in all display locations. However, the video gaming advantage was seen specifically in areas where non-experts performed less well, with higher speed visual processing and shorter minimal exposure duration needed to begin perceptual processing. These higher processing speeds in response to training might improve the temporal resolution of attention and allow attention to be moved between focally and peripherally presented stimuli.

What is particularly interesting about deaf individuals is that the altered sensory experience of deafness clearly brings about unique sensory and cognitive changes. However, most deaf persons are also immersed, to some degree, in a visual spatial language such as British Sign Language (BSL), which in itself places altered conceptual and sensory demands on the visual system, quite different to spoken language (see Bavelier et al., 2006 for a review).

During signed language conversation, fluent, signing individuals typically focus on the face of the person signing to them (Siple et al., 1978; Muir and Richardson, 2005; Agrafiotis et al., 2006). Taking the visuospatial nature of signed language into account, signed language is therefore likely to stimulate peripheral vision in a manner extraordinary to spoken language. Indeed, Swisher et al. (1989) demonstrated that deaf adults could understand American Sign Language (ASL) signs using peripheral vision only, between 45° and 61° eccentric to fixation, whereas hearing individuals could not identify large words presented at similar eccentricities. In signed language space is used both topographically and referentially (MacSweeney et al., 2008). Signing space extends from the navel to above the head and “neutral space” is the area in front of the signer's body at mid-lower chest level one where most of the BSL signs occur (British Deaf Association, 1992). The majority of “words” in ASL are produced below eye level (Teuber et al., 1980); therefore, it may be that the inferior field of vision is particularly stimulated by signed language experience.

Familiarization with visuospatial language does not seem to produce the same enhancements in peripheral vision as revealed in deaf adults: the visual advantages cited earlier in deaf individuals have not been found in hearing signing populations (Neville and Lawson, 1987a,b; Bosworth and Dobkins, 2002; Proksch and Bavelier, 2002; Fine et al., 2005). However, these experiments did not test as far in the periphery as we have tested here. Nevertheless, signing has produced distinct cortical and visual changes. Cortical adaptations have been observed in both hearing and deaf signers in response to language perception. Although a right hemisphere predilection and therefore left VF advantage is widely accepted in the general population (Paillard et al., 1981; Paillard and Amblard, 1985; Clarke et al., 2000), Bosworth and Dobkins (1999, 2002) demonstrated a left hemisphere lateralization and right VF advantage for motion processing in signers, whether deaf or hearing. Bavelier et al. (2001) found early exposure to ASL led to greater reliance on the left hemisphere motion selective area V5/MT. The left, language dominant hemisphere may become increasingly activated by motion processing in deaf and hearing signers, leading to a right VF advantage for the processing of visual motion (Neville and Lawson, 1987a; Bosworth and Dobkins, 1999; Bavelier et al., 2001). Cardin et al. (2013) in an fMRI study of distinct deaf signers and deaf lip readers showed that cortical regions adjust to process the different signals—either speech reading or signed language and that functionally distinct cortical substrates separate deaf adults who sign from those who speech read.

Given the plasticity of the visual periphery to maximize its response to the pattern of visual skills required, one might expect BSL experience itself to influence far peripheral vision and RTs therein. In a previous paper (Codina et al., 2011a) in which we reported deaf and hearing children's peripheral visual performance development on a far peripheral vision task (30–85°), young deaf children (aged 5–8 years) were initially slower to respond to peripheral stimuli than hearing children, they performed similarly at ages 9–11 years, and were significantly faster than controls at ages 12–15 years. To the authors' knowledge, the RT advantage consistently observed in deaf adults has not been thoroughly investigated across the far peripheral field and neither has it been investigated in hearing sign language users. The aim of the current study was to investigate far peripheral visual sensitivity and RT in early onset deaf adults and BSL interpreters, to explore the different and separable effects of auditory deprivation and experience with a visuospatial language.

## Materials and methods

### Participants

All participants were emmetropic; the refractive error did not therefore affect the VF and glasses frames would have interfered with detection of peripheral stimuli. Inclusion criteria for all groups were: good visual acuity in either eye unaided, minimum 0.200 LogMAR units (equivalent to 6/9.5 Snellen acuity, using a standard illuminated ETDRS vision chart at 4 m), absence of epilepsy, and no known abnormal ophthalmological history self-reported during the study consent procedure.

#### Deaf group

Seventeen adults (11 males, 6 females, mean age 33.25 years, range 18–45) with profound binaural hearing loss were recruited by invitation from Grange Crescent Deaf Club in Sheffield, the University of Sheffield, personal contacts, word of mouth through other deaf participants, and from deaf individuals working at Lower Meadow Primary Academy and Allerton Grange School. Criteria for entering the study for deaf participants were: deafness was either present at birth or had onset before the age of 8 months, and was not due to any systemic or genetic disorder known to affect vision such as Usher's syndrome. Eleven participants reported BSL as their native language and 6 reported English. Five participants were left handed and 12 right handed. Nine participants reported being regular action video game players. Four out of the 17 deaf participants contracted deafness as a result of in-uterine rubella and were thus screened by full ophthalmic examination prior to entry into the study to ensure there were no visual deficits.

#### Hearing group

Eighteen participants with no hearing loss and no prior knowledge of any signed language took part in this study (9 males, 9 females, mean age 30.28 years, range 18–45). These participants were recruited through colleagues at The University of Sheffield. Six participants reported themselves as regular action video game players. One participant was left handed and 17 were right handed.

#### BSL interpreter group

Eight participants, all trained and qualified full-time BSL interpreters, registered with ASLI (Association for Sign Language Interpreters), with a minimum of 6 years' experience formed this group (6 females, 2 males, mean age 39.1, range 27–62). Two participants in this group are CODAs and learned sign language as their first language. None of the interpreters reported being action video game players. One participant was left handed and seven were right handed.

#### Eye of testing

Time was a constraint for three participants in the deaf group and two participants in the BSL group who were teachers in one of the schools visited, therefore for these participants only the right eye was tested. For most of the results only the right eye data is presented in line with the work of other authors (Stevens and Neville, 2006; Codina et al., 2011a; Bjerre et al., 2014), as VFs are known to be highly symmetrical in normal subjects (Brenton et al., 1986) and no differences were found between our right and left eye data. We do investigate possible lateralization in the Results section, although the number of participants for which we have data for both eyes is less than that for which we have the right eye data [right eye (*N* = 17), both eyes (*N* = 14) for the deaf group, and right eye (*N* = 8), both eyes (*N* = 6) for the BSL group].

### Stimuli and procedure

**Figure 1:**
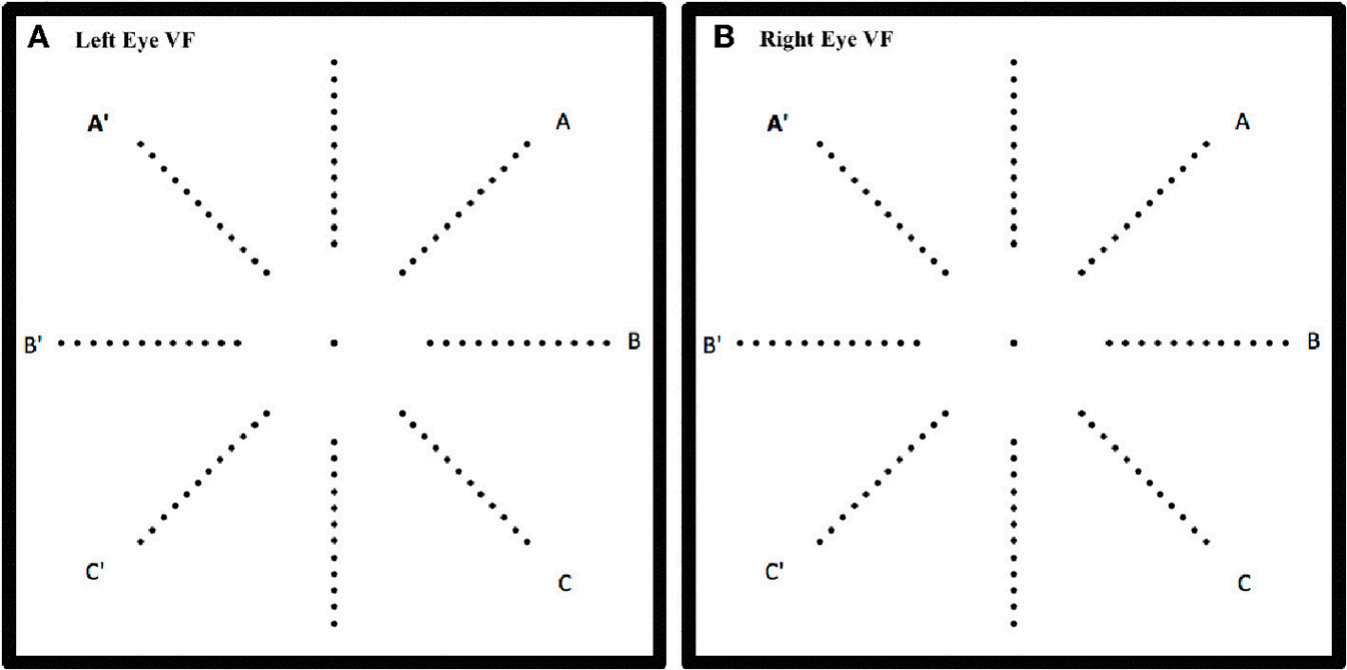
**Schematic of the location of the 96 LEDs in the hemispherical dome on the 8 meridians for left eye (A)** and right eye **(B)**. One of the LEDs was illuminated for 200 ms at a time and the response was recorded only if the participant was fixating the central target.

Informed, written consent was obtained from all participants prior to testing and the study procedures were approved by The University of Sheffield Psychology Department Ethics Committee and complied with the Helsinki Declaration. The methods of this study have been fully described elsewhere (Codina et al., 2011a). In brief, this peripheral vision task was to detect static, briefly illuminated LEDs, presented to the far visual periphery. As is shown schematically in Figure 1 the VF test incorporated 96 LEDs (Nichia, 1.5 cds), implanted into a uniform gray hemisphere (0.5 m radius). This hemisphere contained 12 LEDs along each of the eight meridians that correspond to the four cardinal and four inter-cardinal directions for the right eye and left eye VFs (see Figure 1). The LEDs were positioned between 30° and 85° in 5° steps. An adjustable chin and forehead rest enabled a fixed viewing distance of 1 m and centralization of the participant's eye to the central fixation light behind which was a black and white camera for monitoring fixation. In total 224 LEDs were each very briefly illuminated (for 200 ms) in front of the participant's right eye or left at three different light intensities in a random order. Ninety-six dim stimuli at 83.47 cd/m^2^, 96 medium stimuli at 91.81 cd/m^2^, and only 32 bright stimuli (at 40°, 55°, 70°, and 85° only) of 118.94 cd/m^2^ intensity were presented in a random order. The brightest stimuli were easy to locate and therefore only tested at every third eccentricity to maintain participant interest and check compliance. The data from these stimuli are not reported here. The test was calibrated with an oscilloscope prior to each testing session to ensure uniformity of time period and degree of illumination after transportation. The participant responded by setting a joystick, positioned at chest height, to one of eight possible positions. The joystick was positioned either in front of the right hand or left hand according to self-reported handedness.

The test was carefully explained to each participant in either English or BSL in a lit room and the directions and response directions and instructions for the joystick were both explained and demonstrated to each participant. Participants were seated on an adjustable desk chair, facing the LED array test, their chin and head on rests, aligned and adjusted so that the participant's tested eye was centered 0.5 m behind the fixation target. The other eye was occluded with a patch. All external light sources were eliminated prior to testing and only low level artificial illumination mounted on the upper surface of the hemisphere was provided at a constant level of 1.2 cd/m^2^ brightness for all test environments. Specifically written MATLAB (The MathWorks, Inc.) software with the Data Acquisition Toolbox controlled both the LEDs and logged the data from the joystick via National Instruments data acquisition hardware. Participants first completed a practice trial which consisted of 32 bright stimuli, where four stimuli were presented on every meridian all at eccentricities of 40°, 55°, 70°, and 85°, and on satisfactory completion of the practice the test was begun. Participants were asked to move the joystick to the position corresponding to the meridian of the stimulus LED. Participant fixation during stimulus presentation was observed by the experimenter through a small TV screen monitoring the camera at the fixation point; a stimulus would be repeated later in the sequence if fixation was not maintained. If the participant responded either with the exact matching meridian of the LED or adjacent meridians then this was recorded as a correct response and the RT recorded. We did also record the exactly correct data, when the response exactly matched the LED meridian, and this is only described in the Accuracy data section. Pilot studies had shown that with such peripheral presentations it is difficult to localize the exact position of a flashed LED, particularly for young children. The same procedure was adopted here as we wished to compare our pediatric data (Codina et al., 2011a) with our adult data. All other VF tests reported in the literature used yes/no (detection) responses (Rowe, 2016), and therefore our discrimination paradigm requiring a response accuracy of ±45° was a relatively difficult task.

## Results

### RT data

No differences were found within any test between our right and left eye data; we therefore initially present data from the right eye only, in line with previous authors (Stevens and Neville, 2006; Bjerre et al., 2014). As not all targets were correctly localized by participants, their RT data were analyzed in two separate ANOVAs: by mean meridian RTs averaged across eccentricity and separately mean eccentricity RTs averaged across meridians. Only intermediate and dim stimuli results are presented as the brightest stimuli were used as a control measure.

#### Meridian data averaged across eccentricity

**Figure 2:**
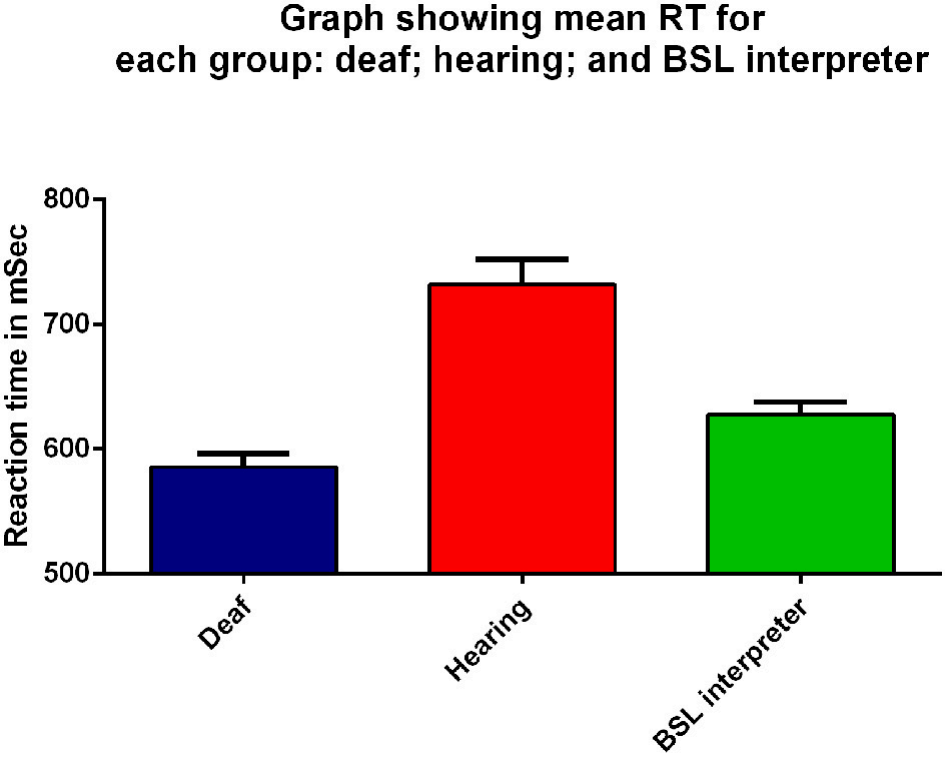
**Mean RT (ms) for all peripheral visual stimuli presented to the right eye for the three groups: deaf (dark blue), hearing (red), and BSL interpreter (green) on the x-axis**. Error bars denote standard error of the mean (SEM). Each group was significantly different from the other two groups (*p* ≤ 0.03) on Bonferroni *post-hoc* analyses.

The mean meridian RT data were analyzed by a three factor mixed measures ANOVA where the factors were group (deaf, hearing or BSL interpreter), stimulus intensity (intermediate and dim) and meridian (8 levels). Figure 2 shows the mean RT data averaged across all stimuli for the right eye for each of the three groups. There was a significant main effect of group [*F*_(2, 40)_ = 4.11, *p* = 0.03]; as can be seen from Figure 2 the mean for the deaf group (mean 585.31 ms) was less than for the hearing group (mean 731.77 ms) with the BSL Interpreter group somewhere in between (mean 627.39 ms). Bonferroni corrected *post-hoc t*-tests showed that deaf adults had significantly faster RTs than either the hearing group (*t* = 6.22, *df* = 33, *p* < 0.001), and the BSL interpreter group (*t* = 2.40, *df* = 24, *p* = 0.03). BSL interpreters also showed faster RTs than hearing non-signers (*t* = 3.29, *df* = 25, *p* = 0.003).

**Figure 3:**
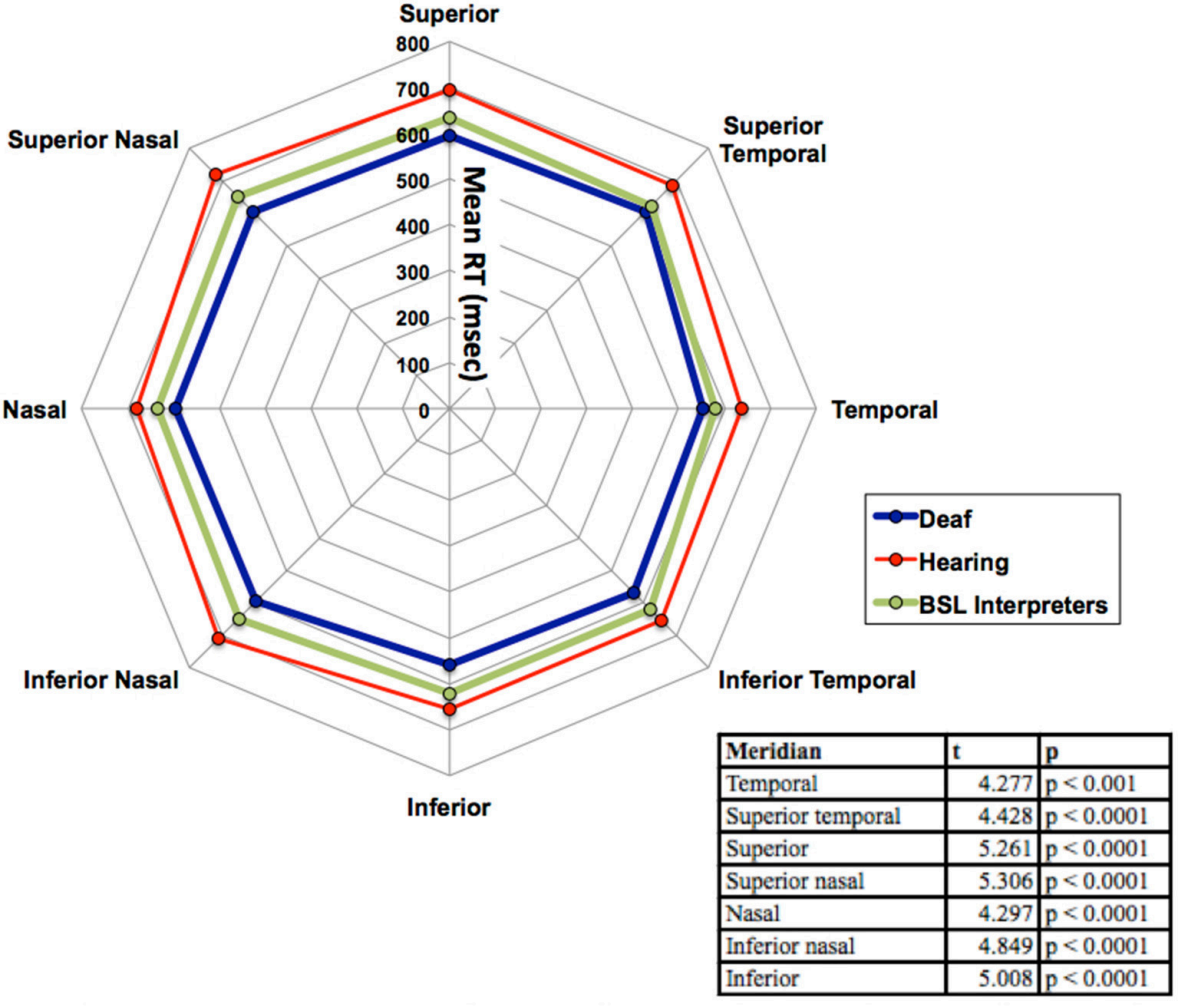
**Mean RT in ms for the three groups (deaf, hearing and BSL interpreter) for the eight meridian locations**. The table within the figure shows the results of the Bonferroni *post-hoc* analyses with *df* = 33 for each reported value. Significant differences were found at each meridian location only between deaf and hearing groups. For clarity, no error bars are shown, but the SEM was between 10 and 17 ms.

There was no significant main effect of stimulus intensity, or any interaction with the other factors therefore all graphs show data averaged across the intermediate and dim stimulus intensities. As can be seen in Figure 3 the mean RTs at each meridian location for the right eye do show some variation and the main effect of meridian was significant [*F*_(7, 280)_ = 17.67, *p* < 0.001], yet the interaction between meridian and group was not significant. No other effects or interactions were significant. Faster RTs are apparent for all three groups in the inferior temporal VF and the hearing and BSL interpreter groups appear closest to each other in this region. Bonferroni corrected *post-hoc t*-tests between groups for each meridian revealed significant differences at each meridian between deaf and hearing groups only and these results are shown in the Table within Figure 3.

#### Eccentricity data averaged across meridian

**Figure 4:**
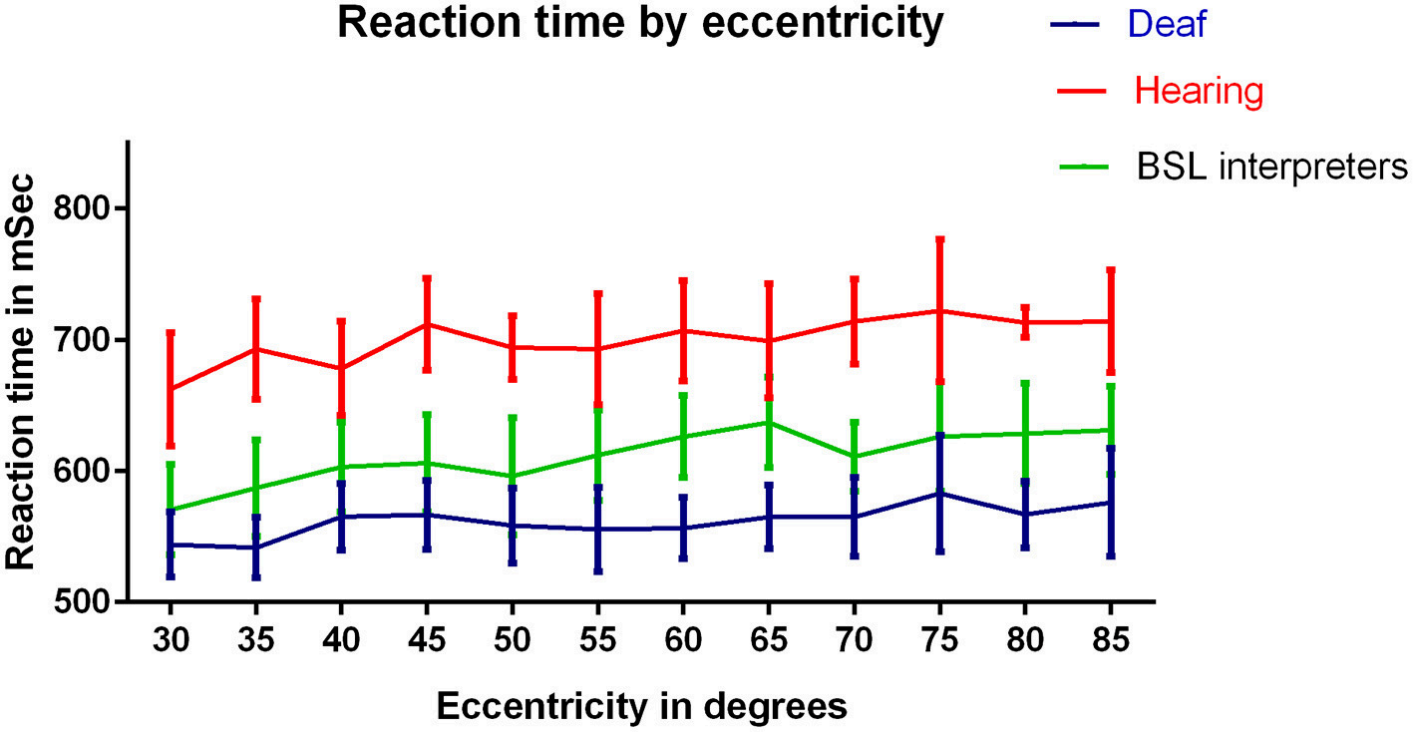
**RTs (ms) for the three groups: deaf, hearing and BSL interpreter for eccentricities tested**. A significant difference between hearing and deaf groups by Bonferroni *post-hoc t*-test was found at eccentricity 70° only (*p* < 0.01). ANOVAs conducted on each pair of groups revealed significant differences between each pairing (*p* < 0.001).

Figure 4 compares mean RTs for eccentricities averaged across meridians for the three groups as a function of eccentricity. A three factor mixed measures [group × stimulus intensity × eccentricity] ANOVA was conducted. There was again, a significant main effect of group [*F*_(2, 40)_ = 3.87, *p* = 0.03]. There was a significant main effect of eccentricity [*F*_(11, 440)_ = 2.28, *p* = 0.01]: increased eccentricity resulted in a slower RT for all three groups. There was a consistent RT ordering of deaf < BSL interpreters < hearing across all eccentricities. There was no interaction between eccentricity and group and no other interaction with eccentricity was significant. However Bonferroni corrected *post-hoc t*-tests showed that deaf adults had significantly faster RTs than the hearing group at 35° (*t* = 2.56, *df* = 33, *p* = 0.02), 60° (*t* = 3.26, *df* = 33, *p* = 0.003) and 70° (*t* = 3.25, *df* = 33, *p* = 0.003), though deaf vs. hearing results were close to significance at most eccentricities.

#### Native language

To determine possible influences of the deaf participants' native language, it was considered as a fourth factor (Native BSL *N* = 11 and native English *N* = 6). The native language factor was not significant, nor did it affect the levels of significance for any other factor. This is an interesting finding, as BSL cannot be wholly responsible for the differences observed in the deaf group. We also tested for differences between action computer game players and non-computer game players within the deaf and hearing groups and found no significant effects or interactions with this factor. However, none of the computer game players would be classed as habitual players under Green and Bavelier's (2003) criteria.

#### Right and left visual fields

**Figure 5:**
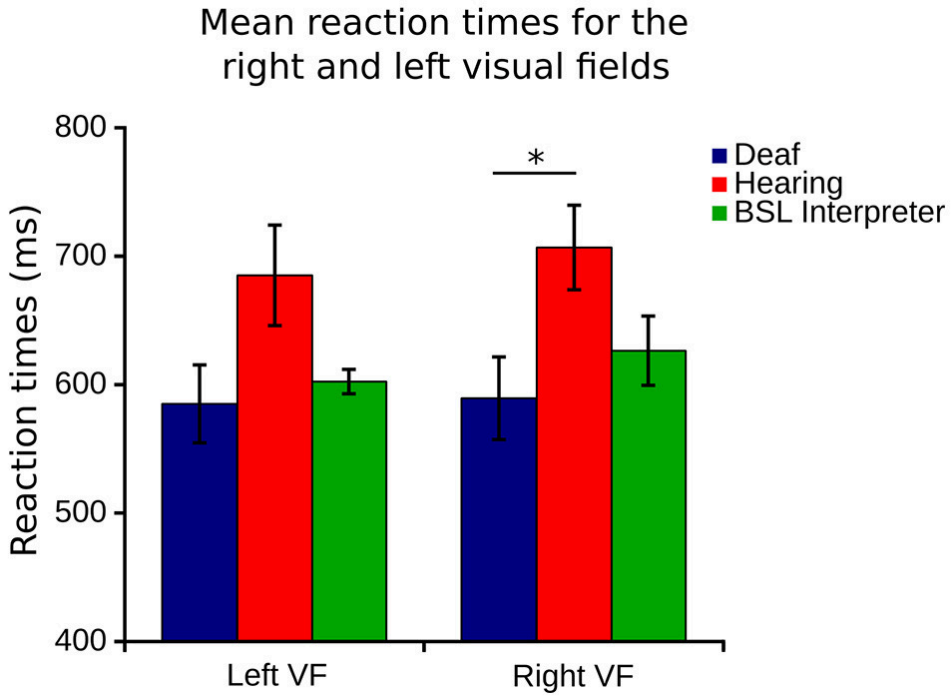
**Right VF (right eye temporal and left eye nasal) and left visual field (left eye temporal and right eye nasal) RT for each of the three groups**. Error bars denote SEM. The capped line and asterisk denotes the significant difference of the bootstrapped Bonferroni *post-hoc t*-test in the right VF between deaf and hearing groups.

Although no differences were found between right and left *eye* data, right and left *visual field* data were different. Lateralization differences have been found before when the right VF (comprised of right temporal and left nasal VFs) is compared to the left VF (left temporal and right nasal). Figure 1 illustrates how the right VF (A,B,C for **both eyes**) and left VF (A',B',C' for **both eyes**) are comprised. Figure 5 shows the mean RT data for each group for this data and a slight left VF RT advantage is observed in all groups. A one way ANOVA with between subjects factor of group (bootstrapped) was conducted on right and left VF data. A significant effect of group was found for the right VF only [*F*_(2, 35)_ = 3.641, *p* = 0.037]. *Post-hoc* pairwise *t*-test comparisons (Bonferroni corrected and bootstrapped) within the right VF showed that deaf participants (*n* = 14, *M* = 589.43, *SE* = 32.2) were significantly faster than hearing (*n* = 18, *M* = 706.8, *SE* = 32.9), (*p* = 0.036). No other differences between groups were significant and no significant differences were found between right and left VFs within any group. Consistent with other published studies (Papadatou-Pastou and Sáfár, 2016) the deaf group had an atypically higher proportion of left handed participants (29% left handed) than the general population, therefore handedness was considered as a second factor in a separate ANOVA. Handedness was not significant, nor did it affect the significance levels of any other factor.

### Accuracy

**Figure 6:**
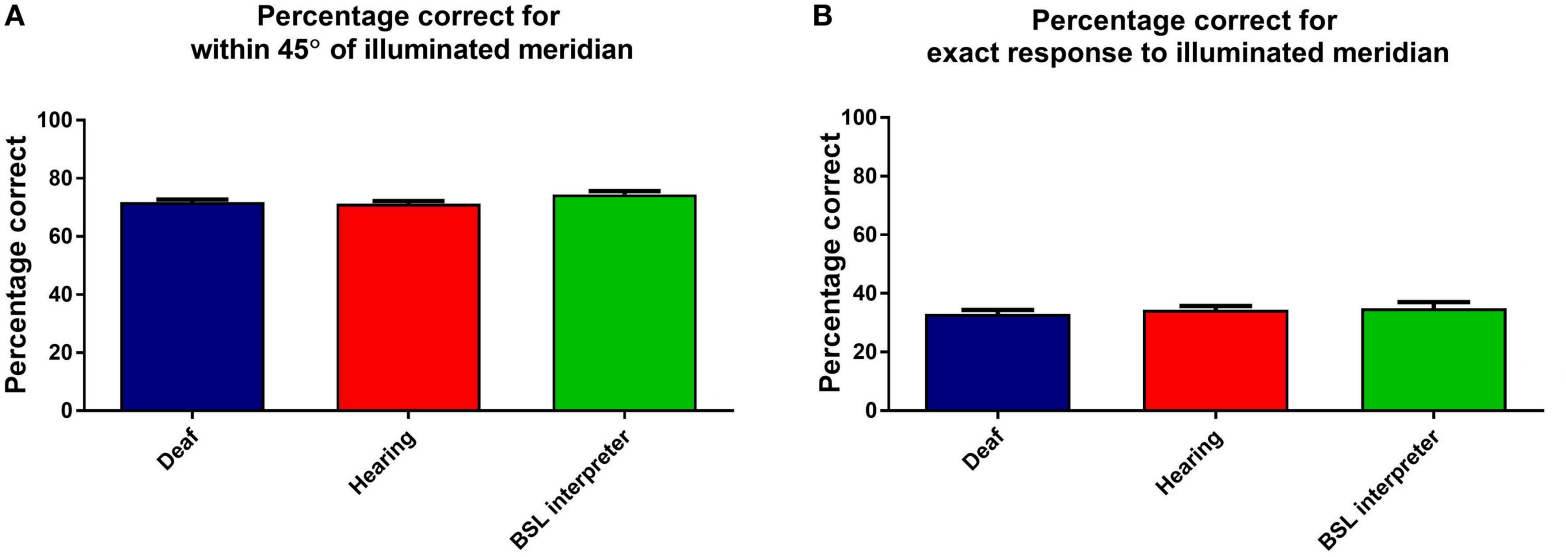
**This Figure shows the mean percentage responses (within 45°) for the three groups: deaf; hearing; and BSL interpreters for (A)** the considered correct response (when within 45° of correct) and **(B)** when the exactly correct meridian was selected. Error bars denote ±1 SEM.

Percentage correct response data were analyzed by a three factor mixed measures [Group × stimulus intensity × meridian] ANOVA. The overall effect of group was not significant, see Figure 6A, which shows that the accuracy is similar for each group. Meridian had a significant effect as expected, due to nasal and superior aspects of the VF being obscured by the nose and brows [*F*_(7, 280)_ = 79.62, *p* < 0.001]. Stimulus intensity had a significant effect [*F*_(2, 62)_ = 6.54, *p* = 0.003], yet there was no significant interaction of group with stimulus intensity and no other significant interactions.

For comparison an identical ANOVA examined data that was only considered exactly correct if the actual correct meridian was chosen by the participant. As expected the percentage of exactly correct responses were found to be lower, yet the levels and factors of significance remained unchanged. Figure 6B shows that the groups also all performed very similarly in the percentage of exactly correct responses.

Overall then accuracy data showed no differences between the groups.

## Discussion

Deaf participants reacted significantly faster to the peripherally presented stimuli when compared to the hearing group and BSL interpreter group. This pattern of results was found across all VF locations and up to the maximum eccentricities tested. Faster RTs in early onset deaf adults found in this study are consistent with the faster deaf RTs reported in the literature (Parasnis and Samar, 1985; Neville and Lawson, 1987a; Loke and Song, 1991; Stivalet et al., 1998; Bosworth and Dobkins, 2002; Proksch and Bavelier, 2002). Importantly, however, the current study demonstrates a greater advantage in the *far* peripheral VF, in a range of peripheral vision (30–85°), which has not previously been investigated. Peripheral visual acuity is increasingly poor at increasing eccentricities, thereby sound confers the greatest advantage at this range of eccentricities. At far peripheral locations our study finds markedly speeded RTs in deaf participants as well as moderately speeded RTs in full time BSL interpreters.

The fastest RTs for all groups and the fastest RTs overall were displayed by the deaf group in inferior and temporal aspects of the VF. One might expect the greatest advantage for deaf individuals in these VF areas where the majority of “words” in signed language would occur (Teuber et al., 1980). However, the significantly speeded RTs were identifiable in all meridian locations and could not be localized to any particular region of the VF. The deaf RT advantage was not significant at all eccentricities, likely because of the increased standard error in eccentricity data. The significant differences in all Figure 4 *post-hoc* analyses revealed differences only between deaf and hearing groups and not between BSL and hearing or between BSL and deaf groups. This deaf advantage at these far eccentricities is consistent with Swisher et al.'s (1989) finding that deaf adults could reliably identify ASL signs 45° and 61° eccentric to fixation using peripheral vision only and with Buckley et al. (2010) report of significantly larger VFs in deaf adults, using kinetic stimuli at a similar range of eccentricities. Buckley and colleagues found that the areas of most significant increase were the inferior and temporal aspects of the VF—regions most stimulated by signed language but also the most expansible aspects of the VF. In deaf adults the RT advantage results from the combined effects of auditory deprivation and the cross-modal plasticity evidenced to this (Fine et al., 2005), as well as immersion into an entirely visual language. However, our results suggest that visuo-spatial BSL language immersion alone does not confer the same peripheral vision RT advantage that auditory deprivation does.

The visuo-cognitive influences of auditory deprivation and signed language exposure are likely to be distinct, yet segregating one from the other is difficult. As previously described, 11 of our deaf group reported BSL as their native language, yet analyses by native language showed no significant influence of native language on RT. However, even in the minority of predominantly aural deaf individuals (for example those married to hearing persons) cumulative exposure to sign language throughout life is still significant. Cardin et al. (2013) reported that after plastic reorganization in deafness, cortical regions adapt to process the different types of signal—either lip reading or signed language—and that functionally distinguishable substrates are present at the cortical level between deaf who sign and deaf who lip read.

Interestingly, Emmorey et al. (2009) found an eye gaze fixation pattern difference between beginning and native signers: beginning signers fixated nearer to the signer's mouth so to perceive lip mouthings more clearly; whereas fluent signers fixated nearer to the interlocutor's eyes. Thus increased experience with signed language was related to a greater ability to perceive signed and mouthed information more peripherally. In our data, the hearing signers were all currently working BSL interpreters, having been fluent in BSL for a minimum of 6 years. They might have therefore adapted during the course of their BSL careers to move further away from lower face fixation as peripheral vision adapts to improve sensitivity to the most peripheral areas most utilized by signed language and facial expression.

In a previous paper (Codina et al., 2011b), we reported that the retinal nerve fiber layer in the eyes of early onset deaf adults was differentially distributed to support peripheral vision, particularly temporal aspects of the VF where the left and right VFs do not overlap and neural resources may be most influential. Fine et al. (2005) reported that the cross-modal plasticity within the auditory cortex responding to signed language was not present in either non-profoundly deaf individuals nor present in hearing signers. The results showing that facilitating this level of neural reorganization requires a dramatically altered sensory experience. That said, it is perhaps only in profoundly deaf adults that increased neural circuitry to the remaining senses is expected, consistent with Fine et al. (2005) and Codina et al. (2011b), which might facilitate the RT decrease identified in the far periphery in this study. However, electrophysiological (Osorio et al., 2010) and brain imaging studies (Ballesteros et al., 2013) have found altered neural correlates in response to simple behavioral training in conceptual object priming, and this in itself may be evidence of compensatory neural activity. Our results are comparable with Buckley et al. (2010) where habitual video game players showed enhanced areas of peripheral visual sensitivity in comparison to non-video-game players, which were even larger in early onset deaf adults. This suggests that enhancement of peripheral vision may be partially mediated by visual attention, with additional compensatory improvement due to sensory deprivation.

Based on our previous research, it is likely that the reduced RTs identified in deaf adults were slow to develop (Codina et al., 2011a), and were perhaps facilitated by altered neural substrates and compensatory neural activity (Codina et al., 2011b). It is possible that similar neural changes may have occurred in hearing signers as well, although this has not yet been tested. However, the visual differences found between hearing signers and non-signers might be similar to the differences found between athletes and non-athletes, not so much in the “hardware” of functional visual pathway changes, but in the “software” efficiency of perceptive and attentional processes (Abernethy et al., 1994; Muiños and Ballesteros, 2013).

Recruitment of BSL interpreters was particularly difficult due to the national shortage of BSL interpreters at the present time (McAleer, 2006), and as a consequence, the mean age of the BSL interpreter group is slightly higher than for deaf and hearing groups. However, simple RT is known to increase with age (Der and Deary, 2006) and become more variable (Hultsch et al., 2002). Also of note was that none of the BSL interpreter group played computer games, as computer game playing has been shown to improve peripheral vision (Green and Bavelier, 2003; Buckley et al., 2010). Therefore, to find faster RTs in this slightly older and non-computer game playing group is a more striking result.

The RTs we report in our study are larger than those studies employing standard kinetic perimetry (Grobbel et al., 2016). Grobel and colleagues, with varying ages of adult participants, reported RTs of 391—522ms. However, theirs was a motion detection task, more suited to the peripheral visual pathway and required the simple press of a button, whereas our experiment utilized static eccentric stimuli and an 8-alternative forced choice task and was therefore conceptually more demanding.

All groups showed a slight left VF RT advantage, in line with the right hemisphere predilection for visual-spatial activity, widely reported in the literature (Paillard et al., 1981; Paillard and Amblard, 1985; Clarke et al., 2000) and reported in hearing non-signers by Bosworth and Dobkins (1999, 2002), Neville and Lawson (1987b). In a stochastic motion task within 15° of fixation, Bosworth and Dobkins (2002) reported that both deaf and hearing signers displayed the opposite pattern of results to hearing non-signers, finding a right VF RT advantage in deaf and hearing signers. We did not find this right VF advantage in the far periphery tested in our study. However, the significant RT reduction in the deaf group's right VF, in comparison with hearing controls, and the highly similar right and left VF RTs in our deaf group may indicate a sensory compensatory mechanism to advance the typical left RT advantage additionally to the right VF. Therefore, auditory deprivation rather than BSL exposure seems to influence the right VF RT. This is interesting in that lesion and neuroimaging studies have consistently reported that the neurobiology of signed language is very similar to spoken language, principally recruiting the left lateralized perisylvian network no matter which language is involved (MacSweeney et al., 2008).

In light of the markedly reduced RTs for the deaf group it is interesting to consider which particular aspects of visuomotor processing may be enhanced by auditory deprivation and training. Auditory deprivation may speed peripheral perception by use of compensatory cortical plasticity and exposure to a language stimulating the visual periphery may call into play alternative visual attention allocation strategies which may, in turn speed the visuomotor response. When considering the visuomotor nature of this study's task it is of note that the deaf group contained a high number of left handed individuals and this finding is consistent with other studies (Papadatou-Pastou and Sáfár, 2016). Atypical handedness may contribute to VF laterality differences, though had no significant effect on our results. Bavelier et al. (2006) put forward four hypotheses in relation to deaf neural and attentional adaptations. They proposed that adaptation may be genetic; that areas V1 and V2 may be more susceptible to intramodal recruitment to visual attention; that multisensory associative cortical areas might reorganize to the remaining modalities such as vision; or that auditory cortex might reorganize to mediate other functions such as vision. Our results suggest that the most speeded responses, highlighting the highest visual attention in the far periphery in deaf adults, supersede the increased visual attention brought about by practice with a visual-spatial language alone, signifying different mechanisms of visual compensation.

## Conclusion

Deaf adults demonstrated significantly faster RTs than both hearing non-signers and hearing BSL interpreters to a range of far peripheral briefly presented static stimuli and this advantage was consistent across all VF locations up to 85° eccentric to fixation. BSL interpreters displayed faster RTs than hearing non-signing adults across the entire VF. Early onset deafness leads to visual compensation in the form of much faster peripheral vision RTs consistent with the cross-modal plasticity benefits to vision of auditory deprivation and use of a visual spatial language. The deaf RT advantage is most apparent in the right VF, where hearing responses are significantly slower. Fluency in BSL without deafness also leads to rapid responses to peripheral stimuli, although not to the same degree as deafness. Daily immersion in a visual-spatial language benefits visual responsiveness to stimuli in the peripheral VF.

## Ethics statement

Ethical approval was granted by the University of Sheffield, Department of Psychology, Ethics Committee. Participants were invited by the experimenter by letter, email or by personal invitation to take part. The researcher gave every participant a participant information sheet that had been given ethical approval. This information was additionally given to the deaf participants in British Sign Language as required. Participants gave full written, informed consent before taking part in the experiment. Deaf adults were given the information verbally, in writing and in British Sign Language as each preferred. Participants were fully informed of the procedure in the language of their choosing. Participants all knew the test procedure and had the opportunity to ask questions under conditions of full lighting before any testing began.

## Author contributions

DB, OP, and CC designed the test equipment, were involved in piloting and refining the experiment and in the ethics process. CC collected and analyzed the data. AL and HB assisted in the interpretation of the data and further analysis. The paper was written by CC with contributions from all authors.

### Conflict of interest statement

The authors declare that the research was conducted in the absence of any commercial or financial relationships that could be construed as a potential conflict of interest.
